# Multiple Ileal and Colonic Stenoses: Is It Always Crohn’s Disease?

**DOI:** 10.1093/ecco-jcc/jjae174

**Published:** 2024-12-16

**Authors:** Sarah Bencardino, Mariangela Allocca, Federica Furfaro, Ferdinando D’Amico, Tommaso Lorenzo Parigi, Silvio Danese, Alessandra Zilli

**Affiliations:** Department of Gastroenterology and Endoscopy, IRCCS Ospedale San Raffaele and Vita-Salute San Raffaele University, Milan, Italy; Department of Gastroenterology and Endoscopy, IRCCS Ospedale San Raffaele and Vita-Salute San Raffaele University, Milan, Italy; Department of Gastroenterology and Endoscopy, IRCCS Ospedale San Raffaele and Vita-Salute San Raffaele University, Milan, Italy; Department of Gastroenterology and Endoscopy, IRCCS Ospedale San Raffaele and Vita-Salute San Raffaele University, Milan, Italy; Department of Gastroenterology and Endoscopy, IRCCS Ospedale San Raffaele and Vita-Salute San Raffaele University, Milan, Italy; Department of Gastroenterology and Endoscopy, IRCCS Ospedale San Raffaele and Vita-Salute San Raffaele University, Milan, Italy; Department of Gastroenterology and Endoscopy, IRCCS Ospedale San Raffaele and Vita-Salute San Raffaele University, Milan, Italy

**Keywords:** Stricture, Crohn’s Disease, differential diagnosis, stenoses

## Abstract

A 62-year-old woman presented with multiple ileal and colonic stenoses, initially suspected to be Crohn’s disease. Despite unremarkable endoscopic biopsies, surgery was performed due to clinical deterioration, and histological analysis confirmed the presence of metastatic breast cancer. This case highlights the importance of considering metastatic disease in the differential diagnosis of gastrointestinal (GI) strictures, particularly when inflammatory bowel disease markers are inconclusive or marginal. Clinicians should be aware of the potential for breast cancer to metastasize to the GI tract, which may present with symptoms mimicking primary GI diseases.

## Graphical Abstract

**Graphical Abstract:**
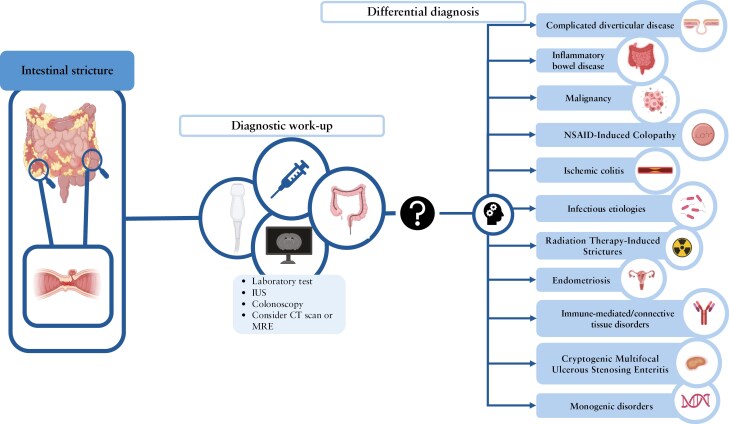

## 1. Introduction

Crohn’s disease (CD) represents a complex challenge in gastrointestinal (GI) medicine due to its chronic inflammation and potential for complications like strictures.^1^ Differential diagnosis is crucial as several conditions can mimic CD, particularly those causing intestinal strictures, including intestinal tuberculosis,^2^ infiltrative diseases (amyloidosis^3^ or sarcoidosis^4^) or malignancies such as metastases from distant cancers.^5^

Metastatic tumors to the GI tract, although rare, can manifest as strictures and may mimic CD clinically and radiologically.^6^ Common sources of metastases include melanoma, breast, lung, and renal cell carcinomas, which can involve the bowel through direct extension or hematogenous spread.^6^ Distinguishing CD from metastatic lesions often requires a comprehensive diagnostic approach integrating clinical history, endoscopic evaluation, histopathology, and imaging modalities such as computed tomography (CT) and magnetic resonance enterography (MRE).^5^

Accurate differentiation is essential as treatment approaches and prognoses vary significantly between CD and metastatic disease. While inflammatory bowel disease (IBD) typically requires lifelong management aimed at controlling inflammation and preventing complications, metastatic lesions necessitate tailored oncological therapies addressing primary tumor control and systemic disease burden.^7^ Therefore, a meticulous diagnostic strategy is fundamental to ensure appropriate management and optimal outcomes for patients presenting with intestinal strictures and suspected underlying pathology.

## 2. Case Report

A 62-year-old Italian woman with ileocecal and multiple colonic stenoses was referred to our IBD outpatient clinic in August 2022 for suspected CD. On physical examination, the patient complained of a weight loss of 10 kg in 2 months and abdominal pain with vomiting and constipation. Initial laboratory evaluation revealed an elevated white blood cell count (12 × 10^9^/L), anemia with hemoglobin of 10 g/dL, a normal creatinine and liver function, a high C-reactive protein of 10 mg/L (reference range <7.4 mg/L), and a fecal calprotectin of 139 µg/g.

**Figure 1:**
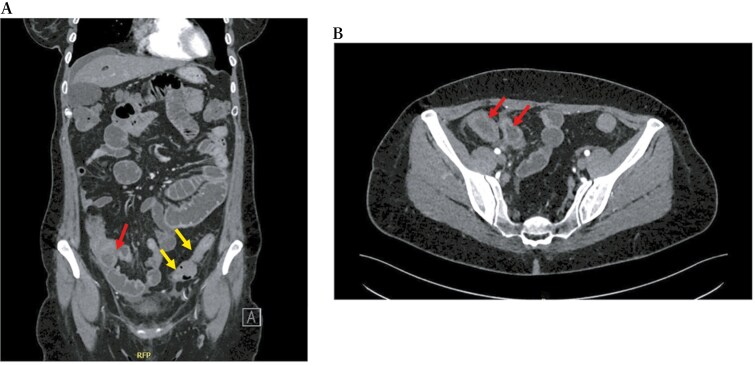
Computed tomography scan, in coronal (A) and transversal (B) sections, shows both a thickening of the last ileal loop for 5 cm in length with enhancement in the arterial phase (red arrows) and a concentric parietal thickening of the sigmoid colon (yellow arrows).

**Figure 2:**
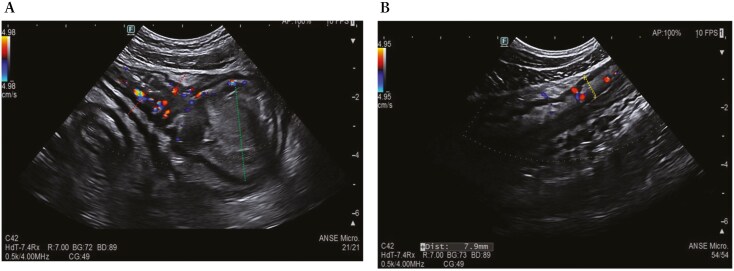
Intestinal ultrasound shows thickening of the terminal ileum (red dotted line) with increased vascularization and upstream dilation (green dotted line) (A) and parietal thickening of the sigmoid colon up to 7.9 mm (yellow dotted line) with increased vascularization in the submucosa and narrowing of the lumen (B).

**Figure 3:**
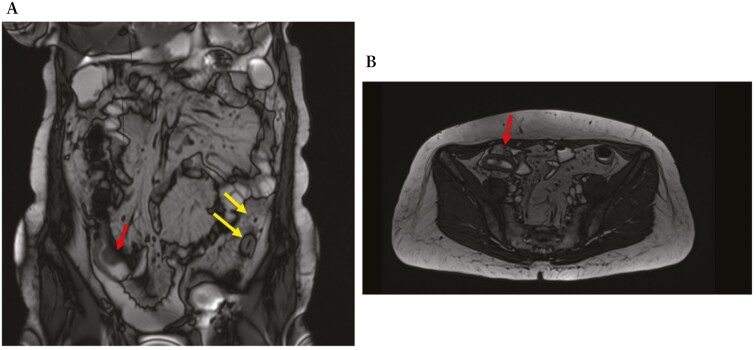
Magnetic resonance enterography, in coronal (A) and transversal (B) sections, shows concentric stricture with the enhancement of the ileocecal valve and terminal ileum (red arrows) and stricture of the sigmoid colon extending over a length of 7 cm (yellow arrows).

**Figure 4:**
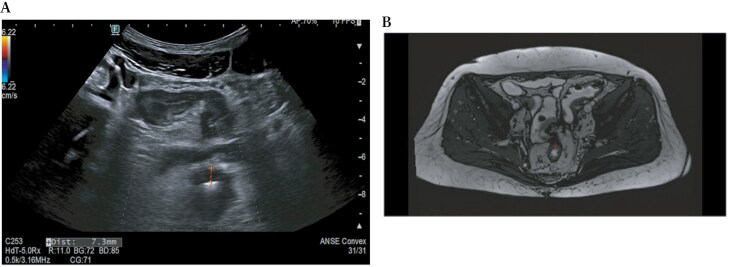
Intestinal ultrasound (A) and magnetic resonance enterography (B) show a wall thickening of the rectum up to 7.3 mm (red dotted lines).

During a recent hospitalization, a CT scan was performed, showing both a thickening of the terminal ileum extended for around 5 cm with enhancement and free fluid around it, and another concentric parietal thickening of the sigmoid colon (Figure 1). This scenario was confirmed by both intestinal ultrasound (IUS) (Figure 2) and MRE (Figure 3). These 2 imaging modalities also revealed a focal wall thickening of the rectum up to 7 mm with the hypoechoic pattern at IUS (Figure 4). Moreover, a QuantiFERON-TB Gold+ test resulted negative suggesting against tuberculosis.

**Figure 5:**
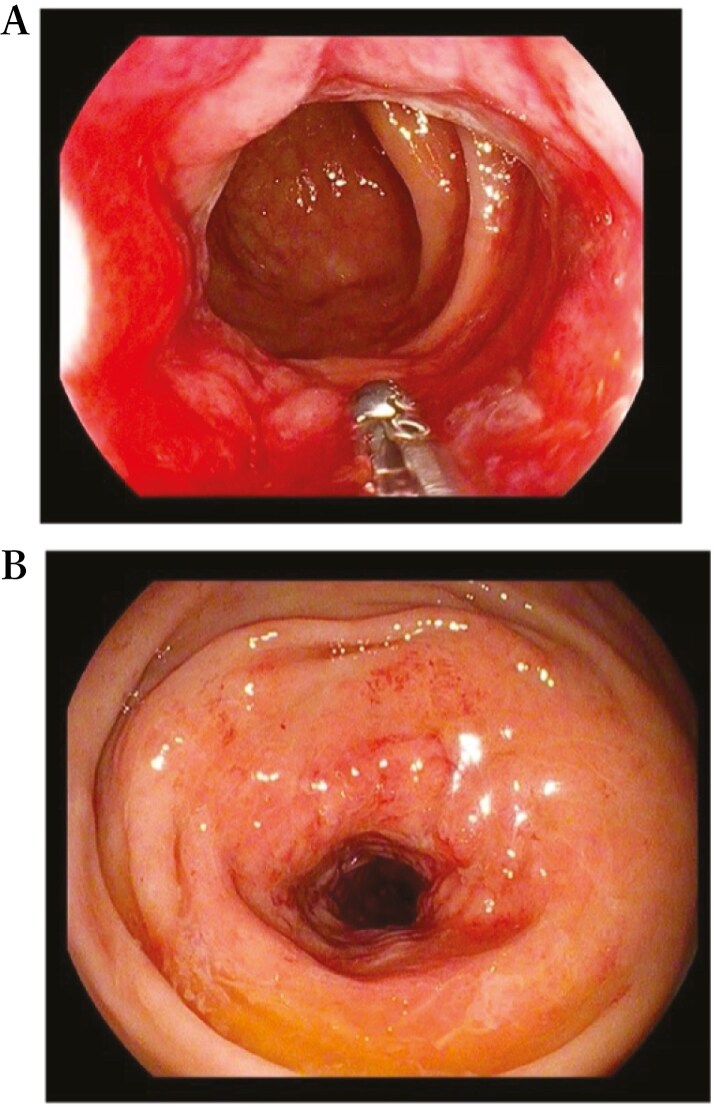
Endoscopic view of a passable stenosis of the rectum extending from 12 to 10 cm from the anal verge.

**Figure 6:**
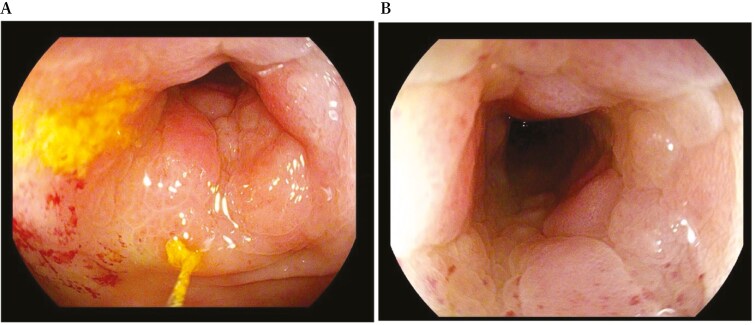
Endoscopic view of a non-passable stenosis of the sigmoid colon at 30 cm from the anal verge.

In accordance with European Crohn’s and Colitis Organization (ECCO) guidelines,^7^ the patient underwent colonoscopy, which confirmed the presence of a non-passable stenosis in the sigmoid colon at 30 cm from the anal verge (not passable even by gastroscope of 9.8 mm diameter) (Figure 5) and a passable stenosis of the rectum extending from 12 to 10 cm from the anal verge (Figure 6), both covered by hyperemic mucosa. Histology showed nonspecific inflammation without dysplasia.

Then the patient was admitted due to clinical deterioration with fever and vomiting and antibiotic therapy was started. After multidisciplinary discussion, surgery was performed (anterior resection of the rectum with Knight-Griffen anastomosis + ileocecal resection + terminal ileostomy). Histological analysis of the all-surgical specimens was positive for breast lobular adenocarcinoma (BLAC) with its typical markers (GATA+, CDX2−, ER = 90%, PgR = 90%, Ki67 = 11%, c-erb-B2 = 0), infiltrating intestinal layers from the serosa to the submucosa. Mammography revealed a 6 cm inhomogeneous area in the left quadrant, and an echo-guided fine needle biopsy was performed: histology confirmed the presence of BLAC, grading G2. The staging CT was negative for further metastases; ribociclib (CDK4/6 inhibitor) and letrozole were started. At 1-year follow-up, CT showed stable disease, and at the last oncological visit in June 2024, the patient was in good clinical condition with good tolerance to therapy.

## 3. Discussion

Metastatic tumors to the GI tract, although rare, can manifest as strictures and may mimic CD.^6^ In this case, clinical, biochemical, and imaging data suggested a diagnosis of CD, but the histological findings of endoscopic specimens did not confirm this hypothesis. The biochemical profile did not align with severe colonic CD, which would be anticipated given the extent of the strictures observed on imaging. While C-reactive protein was mildly elevated, a fecal calprotectin level of 139 µg/g is notably inconsistent with an inflammatory stricture in the distal colon caused by CD, although it is still compatible with fibrostenotic CD. Another factor that argues against the diagnosis of CD is the observation of only minimal inflammation at the site of the non-passable stricture of the sigmoid colon. These discordances are indeed a crucial point for clinicians to consider, as it highlights the importance of integrating biochemical markers with imaging, endoscopy, and clinical presentation.

In cases of stricturing bowel disease that mimic CD, a comprehensive discussion of differential diagnoses is critical. While CD is often the first consideration in patients presenting with bowel strictures, it is important for clinicians to maintain a broad differential, as a variety of other conditions can present with similar clinical and radiological features (Table 1). Recognizing key distinguishing features can guide appropriate management and prevent misdiagnosis.

**Table 1:** Table summarizing the differential diagnoses of stricturing bowel disease that can mimic CD, along with key distinguishing features.

Differential diagnosis	Key features	Distinguishing clues
Malignancy (eg, metastatic breast, lung, melanoma)	Rapid progression of symptoms, atypical location of strictures (eg, distal colon), poor response to IBD therapies.	History of cancer, imaging findings inconsistent with inflammatory disease, rapid clinical worsening with occlusive symptoms, biopsy showing malignancy.
Infectious etiologies (eg, TB, CMV, Yersinia)	Systemic symptoms like fever, night sweats; common in regions with high infection prevalence.	Positive serology, stool tests, cultures, or PCR for specific pathogens; improvement with antimicrobial treatment.
Immune-mediated/connective tissue disorders	Multisystem involvement (eg, skin, joints, lungs); may cause strictures with variable grades of inflammation.	Positive autoantibodies (eg, ANA, ANCA), complement abnormalities, associated symptoms of lupus, vasculitis, or sarcoidosis, and response to immunosuppressive therapy.
Cryptogenic Multifocal Ulcerous Stenosing Enteritis (CMUSE)	Rare; characterized by recurrent small bowel ulcerations and strictures without systemic inflammation.	It typically presents with small bowel strictures, minimal systemic inflammation, endoscopic findings of isolated mucosal ulcers, and normal inflammatory markers.
Monogenic disorders (eg, SLCO2A1-related disease)	Early-onset disease, family history of similar conditions, unresponsive to conventional IBD therapies.	Genetic testing to detect mutations in SLCO2A1 or other relevant genes; typically occurs in younger patients.
Ischemic colitis	Segmental involvement, commonly at watershed areas (splenic flexure, rectosigmoid junction).	Acute onset with abdominal pain followed by bloody diarrhea, typically in older patients with cardiovascular risks. Segmental thumbprinting, bowel wall thickening, or pneumatosis at imaging.
Non-Steroidal Anti-Inflammatory Drug (NSAID)-induced colopathy	Diaphragm-like strictures, more common in the small bowel.	History of long-term NSAID use, absence of transmural inflammation.
Diverticular disease-associated stricture	Localized strictures, usually in the sigmoid colon with pericolic fat stranding.	History of recurrent diverticulitis, no perianal disease, or systemic symptoms. Imaging shows localized thickening, and biopsies reveal fibrosis without granulomas.
Endometriosis	Bowel involvement primarily on the serosal surface.	Women of reproductive age, cyclical symptoms worsened during menstruation. Endometrial tissue on the serosal surface in surgical specimen, not typically seen in endoscopic biopsies.
Radiation therapy-induced strictures	Symptoms may arise months to years post-radiation; fibrosis and scarring in irradiated bowel segments.	History of radiation therapy; stricture typically localized to irradiated area; poor response to anti-inflammatory therapy.

This table provides a concise overview of the different conditions that can mimic Crohn’s disease and their distinguishing clinical, laboratory, and imaging characteristics. Abbreviations: CD, Crohn’s disease; CMV, cytomegalovirus; IBD, inflammatory bowel disease; TB, tuberculosis.

When endoscopic biopsies are negative, suggesting the absence of pathology in the superficial mucosal layers, infiltrative diseases involving the deeper submucosal layers such as amyloidosis^3^ or sarcoidosis^4^ should be considered. Infectious etiologies such as tuberculosis,^2^ cytomegalovirus,^8^ and Yersinia^9^ can mimic CD, particularly in areas with high prevalence. Other diseases that can cause bowel strictures are listed in Table 1 and include immune-mediated and connective tissue disorders (eg, systemic lupus erythematosus,^10^ vasculitis,^11^ and sarcoidosis^4^), Cryptogenic Multifocal Ulcerous Stenosing Enteritis^12^ (CMUSE), monogenic disorders such as SLCO2A1-related disease,^13^ ischemic colitis,^14^ Non-Steroidal Anti-Inflammatory Drug (NSAID)-induced colopathy, complicated diverticular disease,^14,15^ radiation therapy,^15^ and endometriosis.^16^ Finally, malignancy should always be considered, especially in elderly patients or those with a history of cancer. Indeed, metastatic disease, particularly from breast, lung, or melanoma, may present as bowel strictures.^5^ Features such as rapid progression of symptoms, atypical location of strictures, or failure to respond to standard IBD therapies should raise suspicion of malignancy.

Invasive BLAC ranks as the second most prevalent histological subtype of breast cancer, comprising 5%-15% of all invasive cases. It is characterized by non-cohesive cells either individually dispersed or arranged in a single-file linear pattern within a fibrous stroma. Typically, invasive lobular adenocarcinoma is linked with lobular adenocarcinoma *in situ*.^17^

To our knowledge, metastatic spread of breast cancer to the GI tract is very rare and it is more likely to occur in invasive BLAC than in ductal adenocarcinoma.^18^ GI involvement may be the first manifestation of metastatic breast cancer, as in this case, or it may represent a recurrence even many years after the diagnosis of the primary tumor. Given their rarity and unique characteristics, the identification of GI metastases from breast cancer is challenging. This is particularly true in breast cancer patients who present GI symptoms such as nausea, vomiting, diarrhea, and abdominal pain, symptoms that may initially be attributed to treatment side effects or peritoneal carcinomatosis, potentially delaying the accurate diagnosis and treatment.^19^

A recent review analyzed 206 cases of BLAC metastasizing to the GI tract.^20^ Specifically, the most affected site is the stomach (125/206, 60%), with the classical clinical presentation of linitis plastica. In this review, the colon was involved in 11% of cases (23/206), and metastasis manifested clinically mostly as stenosis leading to obstructive symptoms (16/23), and in rarer cases as a symptomatic abdominal mass, polyps, and linitis plastica.

In conclusion, it is important that in cases where clinical and radiological evidence suggests CD, but histological findings are inconclusive, other potential differential diagnoses should be taken into account. Clinicians should be aware that bowel strictures may hide metastases, such as colonic involvement from breast cancer. False-negative histology following biopsy at endoscopy is possible, therefore histological diagnosis with surgery should be considered, especially in patients with obstructive symptoms. Management strategies for these cases are not clearly defined. In selected cases, surgical treatment of abdominal symptomatic localization followed by systemic therapy may contribute to better outcomes.

## Data Availability

No new data were generated or analyzed in support of this research.
